# Efficacy of an advanced hybrid closed-loop system in a patient with type 1 diabetes and intellectual disability: a case report

**DOI:** 10.3389/fendo.2025.1611540

**Published:** 2025-07-31

**Authors:** Hidetsugu Taka, Keiji Sugai, Jumpei Shikuma, Ryo Suzuki

**Affiliations:** Department of Diabetes, Metabolism and Endocrinology, Tokyo Medical University, Tokyo, Japan

**Keywords:** type 1 diabetes mellitus, intellectual disability, insulin pump therapy, Schwachmann-Diamond syndrome (SDS), AHCL system, self-management

## Abstract

Recent advances in automated insulin delivery (AID) system have been remarkable. The advanced hybrid closed-loop (AHCL) system made it easier to achieve optimal glycemic targets. According to previous studies, the AHCL system achieves relatively good glycemic control without strict carbohydrate input. These studies suggested that AID systems potentially improve glycemic control among patients with difficulty with self-management. However, few studies have focused on the efficacy of the systems among these patients. We present a case of a 30-year-old woman with type 1 diabetes and intellectual disabilities (full-scale intelligence quotient = 67) due to Shwachman-Diamond syndrome. While using a sensor-augmented pump with the predictive low glucose suspend system, her glycated hemoglobin levels consistently ranged from 9.0% to 10.0%, and she experienced frequent hospitalizations due to diabetic ketoacidosis. Stepping up to a hybrid closed-loop system achieved better glycemic control and prevented hospitalizations due to diabetic ketoacidosis. Finally, the patient achieved optimal glycemic control through the use of the AHCL system. Our case highlights the potential of AID systems in patients with type 1 diabetes and self-management difficulties, including those with intellectual disabilities.

## Introduction

Recent advancements in diabetes treatment have been remarkable. MiniMed 780G, which is an advanced hybrid closed-loop (AHCL) system, became available. Compared with the hybrid closed-loop (HCL) system, the AHCL system has strict target glucose options and a correction bolus function in addition to the automated basal function. This system enables the achievement of 80% time in range (TIR, sensor glucose 70–180 mg/dL) (1, 2). Furthermore, previous studies using the MiniMed 780G AHCL system in type 1 diabetes patients reported that management goals can be achieved with simple and less accurate carbohydrate counting (3) and can be sustained for twelve months (4). Similarly, a protocol in which bolus insulin was not administered for carbohydrate intake under 80 g still yielded a TIR of 67.5% (5). These results suggest that the AHCL system achieves relatively good glycemic control without strict carbohydrate input and potentially improves glycemic control among patients with difficulty with self-management, such as those with intellectual disabilities. However, to our knowledge, few studies have focused on the efficacy of these diabetes management technologies among these patients.

## Case presentation

### Clinical course from the diagnosis of T1DM to the introduction of the HCL system

A 30-year-old woman with type 1 diabetes and intellectual disabilities due to Shwachman–Diamond syndrome (SDS). The patient demonstrates independence in basic activities of daily living (ADL), but experiences difficulties with instrumental ADL. The patient’s full-scale intelligence quotient (FSIQ) was in the extremely low range (FSIQ = 67). She lives with her parents and is not currently employed. She attends outpatient visits accompanied by her mother on each occasion.

The patient was born at 2900 g without perinatal complications. At age 5, she developed an acute upper respiratory infection and presented with thirst and polydipsia one month after the infection. Her random blood glucose level was 278 mg/dL, and her glycated hemoglobin level was 13.7%. She was diagnosed with acute-onset type 1 diabetes and started multiple daily insulin therapy. Her 24-hour urinary C-peptide was 5.5 µg/day at admission. Because she had failure to thrive (-2 SD or less), neutropenia, and exocrine pancreatic insufficiency, she was suspected of having SDS. Bone marrow analysis revealed dysplasia and fatty changes in all three lineages but no increase in blast cells. Chromosome analysis revealed a normal 46XX karyotype. The patient had mutations in SBDS gene (c.258 + 2T > C and c.183_184TA > CT), which are known to be common mutations in SDS (6). On the basis of these findings, the patient was diagnosed with SDS.

**Figure 1:**
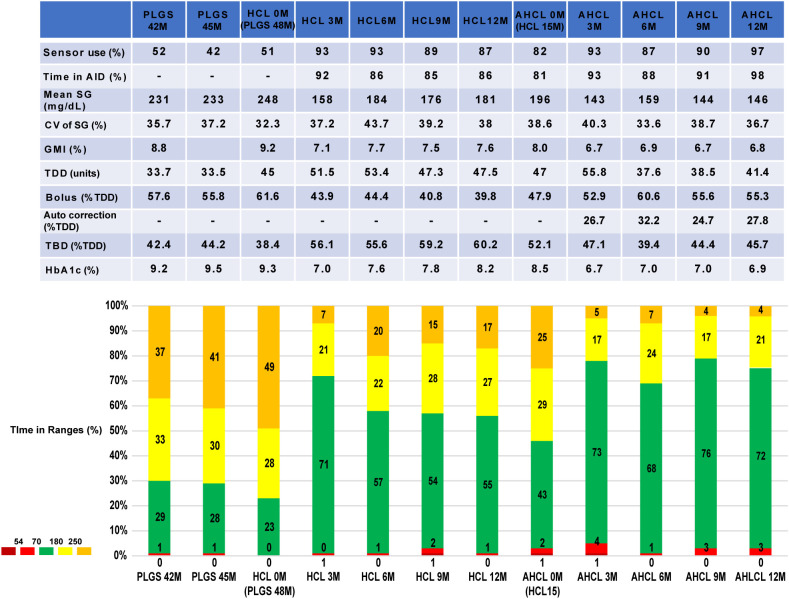
Trends in continuous glucose monitoring parameters. Each CGM metric was calculated according to the CGM targets for adults with type 1 diabetes: TIR 70–180 mg/dL, TAR 181–249 mg/dL, TAR level 2 250- mg/dL, TBR level 1 54–69 mg/dL, TBR level 2 < 54 mg/dL. AID, automated insulin delivery; SG, sensor glucose; CV, coefficient of variation; GMI, glucose management indicator; TDD, total daily dose; TBD, total basal dose.

**Figure 2:**
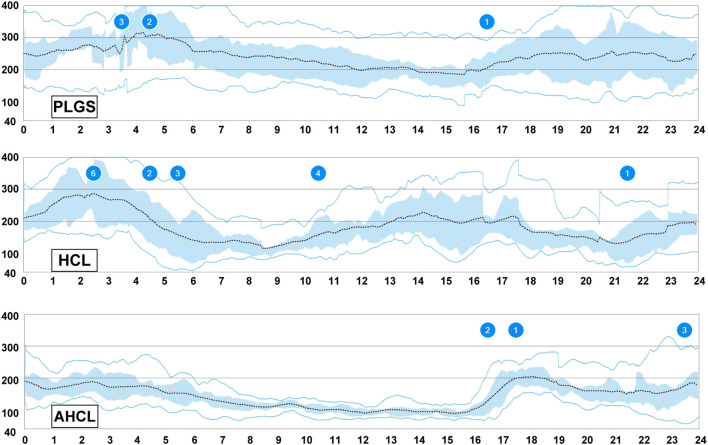
Continuous glucose monitoring graphics under each insulin pump system. The upper panel displays the diurnal glycemic changes under the predictive low-glucose suspend (PLGS) system, the middle panel under hybrid closed-loop (HCL) system (2 months after MiniMed770 initiation), and the lower panel under advanced HCL system (2 months after MiniMed770 initiation). Sensor data were obtained for a period of two weeks. The numbers shown in the graph represent hypoglycemic or hyperglycemic events that are automatically detected by Medtronic’s CareLink system. The high and low glucose values are set at the default levels of 180 and 70 mg/dL, respectively.

At age 19, the patient experienced her first episode of diabetic ketoacidosis and was hospitalized. At that point, her serum and urinary C-peptide levels were below the detection limits. During hospitalization, continuous subcutaneous insulin infusion therapy with use of insulin lispro was introduced, and there were no significant issues with the procedure of attaching an insulin pump. She experienced a second episode of diabetic ketoacidosis and switched to a sensor-augmented pump (SAP) (MiniMed 620G and Enlite Glucose Sensor, Medtronic Inc., MN, USA) at age 23, and then updated to SAP with predictive low glucose suspend (PLGS) systems at age 25. However, her glycated hemoglobin level still ranged from 9.0 to 10.0%, with a high percentage of time above range (**Figure 1**). Hyperglycemia during the night was due to carbohydrate intake without bolus insulin administration or an inappropriate dose of bolus insulin, although she had received nutritional therapy repeatedly. Her blood glucose levels exhibited large day-to-day variations, which complicated the implementation of effective interventions (**Figure 2**). Owing to her increased susceptibility to infections associated with SDS and persistent hyperglycemia, she was prone to developing diabetic ketoacidosis and was hospitalized three times between the ages of 26 and 28.

### Initiation of an HCL system

**Figure 3:**
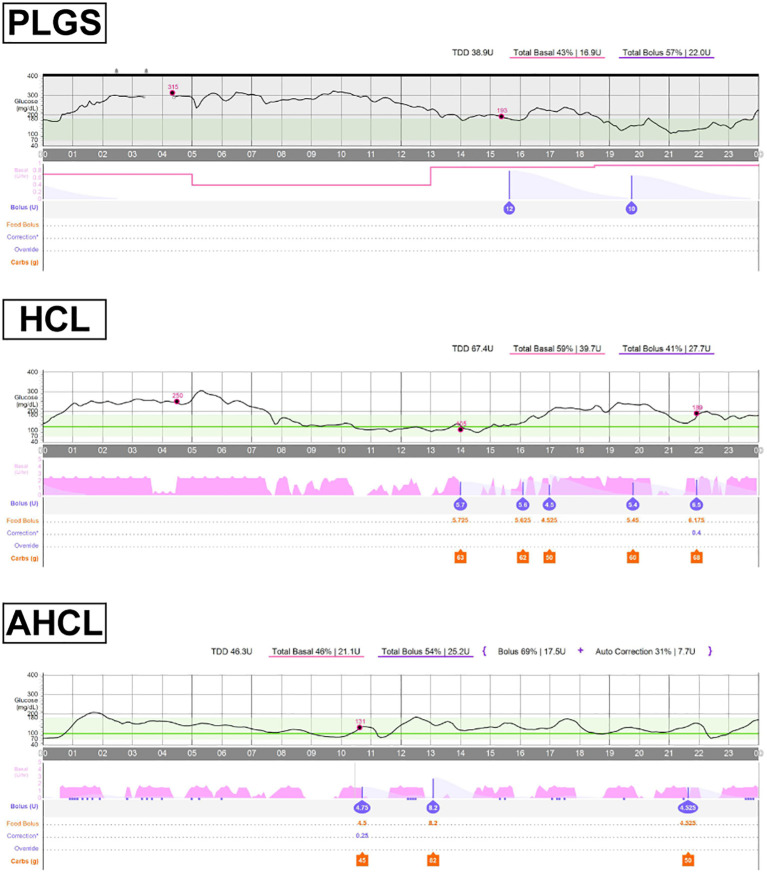
Typical diurnal trends of sensor glucose levels and insulin administration status under the predictive low-glucose suspend (PLGS) system, the hybrid closed-loop (HCL) system, and the advanced HCL system.

An HCL system (MiniMed 770G, Medtronic, Inc., MN, USA) became available in Japan in 2022. MiniMed 770G was equipped with the same HCL system as MiniMed 670G and additionally equipped with connectivity to a mobile application. She started using the system at age 28 when she was hospitalized with diabetic ketoacidosis. The HCL system mitigated her overnight hyperglycemia by increasing the basal insulin and resulting in a glucose level of approximately 120 mg/dL the following morning (**Figures 2**, **3**). With an increase of 10 units or more in the basal insulin administered due to autobasal insulin delivery, the total daily dose (TDD) increased from 30–40 units to 40–50 units. After switching to the HCL system, the proportion of basal insulin increased from 40–50% to 50–60%. Her glycated hemoglobin level decreased to 7.0–8.5% with an increase in TIR to 50–60% (**Figure 1**). However, hyperglycemia was still observed from midnight to 5 AM, likely due to carbohydrate intake without bolus insulin administration (**Figures 2**, **3**). This suggests the limitation of the HCL system in this scenario.

### Initiation of an AHCL system

At age 29, the AHCL system (MiniMed 780G, Medtronic Inc., MN, USA) was introduced in Japan. She started using the AHCL system with the optimized settings: a glucose target of 100 mg/dL and an active insulin time of 2 hours. The automatic correction bolus accounted for 40–50% of the total bolus insulin and improved hyperglycemia at night (**Figures 2**, **3**). Her TIR increased to 70–80% without increasing hypoglycemia, and her HbA1c improved to below 7.0% (53 mmol/mol) two months after starting to use the AHCL system (**Figure 1**). Interestingly, the transition from the HCL system to the AHCL system resulted in a reduction in the TDD to 35–45 U/day. This phenomenon was attributable to an increase in the bolus insulin administered, which facilitated a reduction in her basal insulin, leading to a more efficient insulin delivery regimen. Since the introduction of the HCL system more than 2 years ago, she has not been hospitalized for diabetic ketoacidosis.

## Discussion

We report a patient with type 1 diabetes and intellectual disabilities due to SDS who achieved optimal glycemic control using diabetes management technologies, including HCL and AHCL systems.

SDS is a rare autosomal recessive disorder characterized by exocrine pancreatic insufficiency, immune deficiency, bone marrow failure, and skeletal abnormalities, with 13.6% to 24.5% of patients having mild to severe intellectual disabilities (6). Although the relationship between SDS and type 1 diabetes or other pancreatic endocrine disorders has not been clarified, Gana et al. reported a 3.2% incidence of type 1 diabetes in 62 Italian SDS patients, which is 30 times higher than that reported in the general population (7).

In the present case, the primary factor contributing to poor glycemic control was difficulty with self-management due to the intellectual disabilities associated with the SDS. Specifically, the factors that presented challenges in self-management included the administration of bolus insulin at the correct timing and dosage, as well as the inappropriate intake of carbohydrates. These insufficiencies in self-management led to poor glycemic control and multiple episodes of diabetic ketoacidosis. The HCL and AHCL systems corrected hyperglycemia due to insufficiencies, suggesting the efficacy of diabetes technologies in patients with difficulty in self-management due to intellectual disabilities. In the present case, TIR at one year following initiation of AHCL therapy was 72% (**Figure 1**), which did not reach the level typically attained with strict carbohydrate counting (80.1%), but was comparable to that observed with simplified meal announcements employing a three-tier carbohydrate estimation approach (72.9%) (4). Furthermore, the improvement in glycemic control prevented hospitalization due to diabetic ketoacidosis. In the present case, several factors may have contributed to the successful implementation of the HCL and AHCL systems. First, she could equip herself with an insulin pump and continuous glucose monitor (CGM) sensor and maintain appropriate sensor use and time in HCL and AHCL. Among patients who are unable to independently wear the devices, it is crucial to provide sufficient support to ensure proper sensor use and time in HCL and AHCL. Second, appropriate use of an insulin pump requires the ability to respond to alerts and to address issues such as cannula occlusions. In this case, the patient was able to manage these situations independently and did not experience any episodes of diabetic ketoacidosis or severe hypoglycemia requiring hospitalization following the initiation of the HCL and AHCL systems. For patients who are unable to manage these tasks independently, the implementation of AID therapy necessitates the provision of adequate support. Third, parental support played a crucial role as demonstrated in a previous study highlighting the importance of parental support in patients with T1DM and intellectual disabilities (8). The mother of the present patient encouraged the administration of bolus insulin before meals, which resulted in a high TIR during the daytime. Although no quantitative evaluation was conducted before and after the introduction of the AHCL, the mother reported that the introduction of the AID system reduced her psychological burden, suggesting that the use of AID can also be beneficial for caregivers.

In conclusion, we reported a patient with T1DM with intellectual disabilities who achieved optimal glycemic control using an AHCL pump. Our case highlights the potential of AID systems in achieving optimal glycemic control and preventing hospitalizations due to diabetic ketoacidosis in patients with type 1 diabetes and self-management difficulties, including those with intellectual disabilities.

## Data Availability

The original contributions presented in the study are included in the article/supplementary material. Further inquiries can be directed to the corresponding author.
